# Online information about mammography screening in Italy from 2014 to 2021

**DOI:** 10.1186/s12905-022-01718-w

**Published:** 2022-04-27

**Authors:** Francesco Attena, Lucia Abagnale, Angela Avitabile

**Affiliations:** Department of Experimental Medicine, University of Campania “Luigi Vanvitelli”, Via Luciano Armanni, 5, 80138 Naples, Italy

**Keywords:** Website, Breast cancer prevention, Mammography, Information

## Abstract

**Background:**

Many studies have reported that the information women receive about the risk-to-benefit ratio of breast cancer screening is still scarce and biased toward benefit. In a study we conducted in 2014, we analysed online documents about breast cancer screening that were addressed to the general female public. In the present study, we used the same methodology to verify if the information provided to women was improved.

**Methods:**

We evaluated documents addressed to the general female public and posted on the Internet by the Italian national and regional public health services. False-positive and false-negative screening results, biopsy-proven false-positive results, interval cancer, overdiagnosis, radiation exposure, and decrease in risk of mortality were analysed. In addition, quantitative data were searched.

**Results:**

In 2021, the most frequently reported information was reduction in breast cancer mortality (58.2%). The most frequently reported risk was a false-positive mammogram (42.5%). Similar frequency rates were reported for interval cancer, false-negative result, and radiation exposure (35.8%, 31.3%, and 28.3%, respectively). Overdiagnosis and biopsy-proven false-positive result were the less reported risks (20.1% and 10.4%). Thirteen documents provided quantitative data about reduction of mortality risk (16.7%), and only 19 provided quantitative data about risks or harms (8.4%). Almost all organisations sent letters of invitation to women (92.5%) and provided screening free of charge (92.5%). The most recommended was biennial screening for women aged between 50 and 69 years (48.5%). Compared with the information in 2014, that in 2021 showed some improvements. The most marked improvements were in the numbers of reports on overdiagnosis, which increased from 8.0 to 20.1%, and biopsy-proven false-positive result, which increased from 1.4 to 10.4%. Regarding the benefits of breast cancer screening, reduced mortality risk became increasingly reported from 2014 (34.5%) to 2021 (58.2%). Conversely, quantitative data remained scarce in 2021.

**Conclusions:**

Moderate improvements in information were observed from 2014 to 2021. However, the information on breast cancer screening in documents intended for women published on Italian websites remain scarce.

## Introduction

Breast cancer screening is one of the most debated scientific topics, with many issues that are still controversial and divisive. Its usefulness is probably the most important issue because some studies still consider breast cancer screening as a scarcely effective or ineffective tool for reducing breast cancer mortality or to have an unfavourable benefit-to-risk ratio [1–6]. The debate among women is equally intense and mainly concerns the degree of information they receive. Despite the current era of transparent communication and informed consent, many recent studies have reported that the information women receive on the risk-to-benefit ratio of breast cancer screening is still scarce and biased toward benefit [7–11].

Women can obtain information about breast cancer prevention in many ways. When women request or are invited to undergo breast cancer screening, the health operators involved should support them in achieving a shared decision making [12–16]. This informed participation in the decision making implies that health operators must provide women with correct and comprehensive information about the benefits and risks of breast cancer screening to help them decide in accordance with their personal values. When women try to find out about mammography on their own, information about breast cancer screening are accessible from various sources (websites, journals, television, oncological centres, or other health organisations) and is provided in various formats (leaflets, booklets, brochures, pamphlets, and technical reports). Among these sources, Internet-based information on health concerns has become increasingly important.

In a previous study we conducted in 2014, we analysed documents about breast cancer screening specifically addressed to the general female public and posted online by the Italian national and regional health services, local health authorities, and major hospitals [17]. The aim was to evaluate the type and completeness of information about the risk-to-benefit ratio of mammography screening. In the present study, we used the same methodology to verify any improvement in the information provided to women during a 7-year period.

## Methods

We reported below the same methodology of our previous study [17].

### Types of web page

We analysed web pages specifically addressed to the general female public and published by the Italian national and regional health services, local health authorities, or major hospitals. We excluded technical documents specifically directed to health-care personnel but included a few documents addressed to an unclear audience.

### Search strategy

Although Italy has a national health service (Servizio Sanitario Nazionale), each Italian region has its own regional health service (Servizio Sanitario Regionale). Therefore, the organisation of and communication about mammography services vary throughout Italy. Furthermore, each region has local health authorities (Aziende Sanitarie Locali [ASL]), major hospitals (Aziende Ospedaliere [AO]) and local hospitals (Presidi Ospedalieri [PO]) affiliated with ASL. Therefore, our search strategy included national, regional, and local levels hierarchically.

At the national level, we searched official websites of the four main national health institutions, namely Ministero della Salute, Istituto Superiore di Sanita, Agenas (Agenzia Nazionale per i Servizi Sanitari), and Osservatorio Nazionale Screening. For regional and local information, we examined the health services section of the official website of each region and all ASL and AO official websites in each region. The search on all these websites was performed with the Google search engine. We thought this is the main search strategy used by Italian women. We excluded private health organisations, as a complete list was not available and as mammography screening is almost exclusively supported by the SSN.

## Keywords

On each official website, we performed a search using the following terms: mammografia (mammography), prevenzione dei tumori (cancer prevention), tumore della mammella (breast cancer), screening tumore della mammella (breast cancer screening), and prevenzione (prevention). When no documents appeared in the search using these terms, we performed additional searches in the websites.

### Assessment of health information

Two medical researchers, who were residents in public health, epidemiology, and hospital organisation, analysed the websites independently and in a blinded manner to evaluate information on potential risks and harms, including false-positive and false-negative results, biopsy-proven false-positive results (i.e. a false positive on mammography confirmed as false positive even after biopsy), interval cancer, overdiagnosis, and radiation exposure, and on potential benefits, including reduced risk of mortality and increased chance of survival. In addition, quantitative data (e.g. percentages, relative risk reduction, number needed to screen) were searched. After the evaluation for each region, the results were compared, and minor discrepancies were discussed and resolved.

Other collected data included information on whether the examination was free of charge or not (yes/no), justification for absence at work (yes/no), whether a letter of invitation was given to each woman (yes/no), age range, and frequency. The websites were accessed between February and May 2021. Ethics committee approval was not required for this study because it did not involve patients.

## Results

In accordance with the search strategy, we examined 166 websites (Table 1). Among these websites, 134 (80.7%) had web pages addressed to the general female public and 73 (54.5%) reported at least one risk of breast cancer. Therefore, almost half of these sites didn’t give any information about risk/benefit of screening. Overall, the mean number of reported risks in each document was 1.7 (226/134 websites). In 2014, more websites were found (255 vs 166) because after this period, significant mergers occurred between health organisations. However, the number of documents on breast cancer screening that were addressed to women is similar between 2014 and 2021. In general, more documents discussed at least one risk (54.5% vs 43.4%) and more risks were reported in 2021 than in 2014 (226 vs 152).

**Table 1:** Documents that reported information on breast cancer screening and at least one risk of breast cancer screening

	2021		2014	
	n	%	n	%
Web pages searched	166*	–	255	–
Documents addressed to the female public	134	80.7	136	53.3
Documents discussing at least one risk	73	54.5	59	43.4
Total risk reported	226	–	152	–

*4 national, 20 regional, 41 AO, 101 ASL

The most reported information was the reduction in breast cancer mortality (58.2%; Table 2), with false-positive result being the most frequently reported risk (42.5%). Similar rates were reported for interval cancer, false-negative screening result, and radiation exposure (35.8%, 31.3%, and 28.3%, respectively). Overdiagnosis and biopsy-proven false-positive result were the less frequently reported risks (20.1% and 10.4%, respectively). Of the 226 risks reported, only 19 were supported with quantitative data (8.4%), and more than half (11) were overdiagnosis. Thirteen documents had quantitative data about reduced risk of mortality (16.7%).

**Table 2:** Information about the risks and benefits of breast cancer screening by Italian public health organisations in 2021 and 2014

	2021 134 documents			2014 136 documents		
	n	*q*	%	n	*q*	%
Risks						
False-positive result	57	5	42.5	42	4	30.8
Biopsy-proven false-positive result	14	0	10.4	2	0	1.4
False-negative result	42	1	31.3	27	5	19.9
Interval cancer	48	2	35.8	30	3	22.1
Overdiagnosis	27	11	20.1	11	3	8.0
Radiation exposure	38	0	28.3	40	0	29.4
Total risk	226			152		
Total quantitative data on risks		19	8.4		15	9.8
Benefits						
Reduced breast cancer mortality	78		58.2	47		34.5
Quantitative data on Reduced breast cancer mortality		13	16.7		17	36.2

*q* = number of sites that offered quantitative information about the indicated risk or benefit

Compared with the information in 2014, the information in 2021 had moderate improvements for all single items. The most marked improvements were in the numbers of reports on overdiagnosis, which increased from 8.0% to 20.1%, and biopsy-proven false-positive result, which increased from 1.4% to 10.4%. Regarding the benefits of breast cancer screening, reduced risk of mortality was increasingly reported from 2014 (34.5%) to 2021 (58.2%). Conversely, quantitative data remained scarce, with the exception of those for overdiagnosis (11 vs 3).

Almost all organisations sent letters of invitation to women (92.5%) and provided screening free of charge (92.5%). Some women also received justified absence from work (14.2%). The age group recommended for screening was highly variable; the most common was biennial screening.

for women aged between 50 and 69 years (48.5%). However, 34 documents (25.4%) anticipated screening at the age of 45 years; and 2, at the age of 40 years (Table 3).

**Table 3:** General information about breast cancer screening by Italian public health organisations

	n	%
General characteristics		
Documents/websites	134/166	80.7
Justified absence at work	19	14.2
Free-of-charge test	124	92.5
Letter of invitation	124	92.5
Screening programs by age group		
50–69 y.o., biennial*	65	48.5
50–74 y.o., biennial	18	13.4
45–49 y.o., annual/50–69 y.o., biennial	1	0.74
40–49 y.o., annual/50–69 y.o., biennial	2	1.5
45–69 y.o., biennial	4	2.9
45–49 y.o., annual/50–74 y.o., biennial	19	14.2
45–49 y.o., annual/50–69 y.o., biennial/70–75 y.o., biennial	10	7.5
No information	45	33.6

y.o. = years old

*Guidelines of Italian Ministry of Health

## Discussion

The level of information provided to women on the benefit-risk balance of breast cancer screening is still low. However, comparing with data collected in 2014, we found some improvements in the information in 2021.

The most frequently reported risk of breast cancer screening was a false-positive result, although not all the reported information was clear. The following are two examples of unclear reporting of false-positive results: 1. ‘If the test is not legible or any changes are detected, the user is contacted directly by the competent staff to repeat it or to carry out further diagnostic tests’ [18]. 2. ‘Positive users will be contacted by telephone by the operators of the Screening Center who will invite them to undergo further tests (2nd level exam, such as clinical examination, ultrasound, MRI, cytological examination, biopsy, etc.).’ In case of further positivity, the patient will be advised on the most suitable treatments for her clinical situation [19].

Information about overdiagnosis showed a notable increase in 2021 compared with 2014. However, the frequency of this information in the documents aimed at women was still low, probably because it is both the most recent and harmful risk for women. Therefore, not all health operators are aware of overdiagnosis, and if they are aware of it, they might avoid reporting the information in public documents for fear of dissuading women from undergoing screening [20, 21]. Moreover, we considered many reports of overdiagnosis unclear. For example, ‘It is also possible that mammography reveals very small but slow-growing tumors (about 10%) that are not harmful for the woman's health’ [22]*.* Considering that overdiagnosis is the most important risk for women, the main scientific efforts on this screening are directed toward reducing overdiagnosis by understanding tumour heterogeneity and how indolent cancers evolve and progress [23].

The persistent lack of information is also a common finding in other countries, both from the website search [8, 24–27] and written documents [9, 28–30]. This seems surprising because overcoming the paternalistic physician–patient relationship has long been universally accepted and, in our case, because the scientific literature has continued over the years to emphasise the importance of an informed choice for women [12–16]. This condition persists for many reasons. One of the most frequently reported justification is that providing information on potential harms could reduce adherence to screening. Scientific literature reports contradictory data, as some studies have shown a reduction in adherence [31], whereas others more often showed that correct information only slightly reduced [36] or did not reduce adherence to screening [7, 32–34]. Whatever the causes of this lack of information for women, the main way to overcome them is the training of operators. If operators are not up to date, they will receive the correct update on the risks/benefits ratio of mammography. If the operators are aware of this ratio, they will receive the correct update to provide, in Italy through informed consent form, women with complete information on the risks and benefits of mammography. However, from an ethical point of view, preventing reduction in adherence cannot justify the lack of information.

Other reasons for the scarce information is the gap between research and practice, which has resulted in many breast cancer practitioners being not well updated about the controversial risk-to-benefit ratio of breast cancer screening [35]. Major propensities towards breast cancer screening, with risk minimisation, may depend on professional interests. Radiologists, breast cancer specialists, and other involved health workers may have distorted judgments due their professions [36]. Then, explaining risk/benefit in a simple and short way takes time and patience for the operators. Furthermore, breast cancer screening is strictly incorporated in women’s struggle for emancipation and thus has become a red flag for criticism, as any criticism is considered by women’s movements as an anti-feminist attack on their health and freedom. This is the paradox of breast cancer screening: for women to be fully in control of their own body, they receive less information on breast cancer screening than on any other health topic.

Much information was unclear perhaps to avoid alarming patients. Instead, a valid explanation is provided for the absence of quantitative information; which is the wide variability of data in the literature and the difficulty of summarising these data in a way that would be accessible to non-experts.

However, our findings could be underestimated as we have investigated only one of the three moments where it is possible to inform women. In addition to active web searching, most italian women receive at home a letter of invitation to undergo screening. Furthermore, if they go for screening, they must sign an informed consent. It is the task of each care facility to best distribute information among these three documents.

Our study has limitations. As just said, one is that it did not analyse invitation letters, brochures or informed consent forms given to women before undergoing mammography, which could contain more information than the corresponding websites. We excluded private treatment centres and did not know whether they offer more or less information than public centres. The comparison between 2014 and 2021 could be biased mainly because the health operators who searched the websites were different.

## Conclusions

In conclusion, our results showed moderate improvements in the information about the risks and benefits of breast cancer screening from 2014 to 2021. However, the documents posted on Italian websites were still lacking, as these did not provide correct and complete information to women who wanted to undergo breast cancer screening, preventing them from making fully informed choices about their health.

## Data Availability

All visited Links are available on request from the corresponding author.

## Declarations

### Approval

The ethics committee approval was not required because the study did not involve any people.
